# Head and neck cancers survival in Europe, Taiwan, and Japan: results from RARECAREnet Asia based on a privacy-preserving federated infrastructure

**DOI:** 10.3389/fonc.2023.1219111

**Published:** 2023-09-13

**Authors:** Laura Botta, Tomohiro Matsuda, Hadrien Charvat, Chun-ju Chiang, Wen-Chung Lee, Anna Jacoba van Gestel, Frank Martin, Gijs Geleijnse, Matteo Cellamare, Simone Bonfarnuzzo, Rafael Marcos-Gragera, Marcela Guevara, Mohsen Mousavi, Stephanie Craig, Jessica Rodrigues, Jordi Rubió-Casadevall, Lisa Licitra, Stefano Cavalieri, Carlo Resteghini, Gemma Gatta, Annalisa Trama, the RARECAREnet working group

**Affiliations:** ^1^ Evaluative Epidemiology Unit, Department of Epidemiology and Data Science, Fondazione IRCCS Istituto Nazionale dei Tumori, Milan, Italy; ^2^ Insititute of Cancer Control, National Cancer Center, Tokyo, Japan; ^3^ Faculty of International Liberal Arts, Juntendo University, Tokyo, Japan; ^4^ Division of International Health Policy Research, Institute for Cancer Control, National Cancer Center, Tokyo, Japan; ^5^ Taiwan Cancer Registry Center, and Institute of Epidemiology and Preventive Medicine, Taipei, Taiwan; ^6^ IKNL, Department of Research & Development, Netherlands Comprehensive Cancer Organisation, Utrech, Netherlands; ^7^ Analytical Epidemiology and Health Impact Unit, Department of Epidemiology and Data Science, Fondazione IRCCS Istituto Nazionale dei Tumori, Milan, Italy; ^8^ Epidemiology Unit and Girona Cancer Registry, Oncology Coordination Plan, Department of Health, Autonomous Government of Catalonia, Catalan Institute of Oncology, Girona Biomedical Research Institute (IdiBGi), Universitat de Girona, Girona, Spain; ^9^ Biomedical Network Research Centers of Epidemiology and Public Health (CIBERESP), Madrid, Spain; ^10^ Group of Descriptive and Analytical Epidemiology of Cancer, Josep Carreras Leukemia Research Institute, Girona, Spain; ^11^ Navarra Cancer Registry, Instituto de Salud Pública y Laboral de Navarra, Pamplona, Spain; ^12^ Epidemiology and Public Health Area, Navarra Institute for Health Research (IdiSNA), Pamplona, Spain; ^13^ East Switzerland Cancer Registry, St. Gallen, Switzerland; ^14^ Patrick G. Johnston Centre for Cancer Research, Queen’s University Belfast, Belfast, United Kingdom; ^15^ Cancer Epidemiology Group, IPO Porto Research Center (CI-IPOP), Portuguese Oncology Institute of Porto (IPO Porto)/Porto Comprehensive Cancer Centre (Porto.CCC) & RISE@CI-IPOP (Health Research Network), Porto, Portugal; ^16^ Medical Oncology Department, Catalan Institute of Oncology, Girona, Spain; ^17^ OncoGirPro Group, Biomedical Research Institute (IDIBGI), Girona, Spain; ^18^ Head and Neck Medical Oncology Department, Fondazione IRCCS Istituto Nazionale dei Tumori, Milan, Italy; ^19^ Department of Oncology and Hemato-Oncology, University of Milan, Milan, Italy

**Keywords:** population-based cancer registry, survival, head and neck cancers, geographical differences, federated learning approach

## Abstract

**Background:**

The head and neck cancers (HNCs) incidence differs between Europe and East Asia. Our objective was to determine whether survival of HNC also differs between European and Asian countries.

**Methods:**

We used population-based cancer registry data to calculate 5-year relative survival (RS) for the oral cavity, hypopharynx, larynx, nasal cavity, and major salivary gland in Europe, Taiwan, and Japan. We modeled RS with a generalized linear model adjusting for time since diagnosis, sex, age, subsite, and histological grouping. Analyses were performed using federated learning, which enables analyses without sharing sensitive data.

**Findings:**

Five-year RS for HNC varied between geographical areas. For each HNC site, Europe had a lower RS than both Japan and Taiwan. HNC subsites and histologies distribution and survival differed between the three areas. Differences between Europe and both Asian countries persisted even after adjustments for all HNC sites but nasal cavity and paranasal sinuses, when comparing Europe and Taiwan.

**Interpretation:**

Survival differences can be attributed to different factors including different period of diagnosis, more advanced stage at diagnosis, or different availability/access of treatment. Cancer registries did not have stage and treatment information to further explore the reasons of the observed survival differences. Our analyses have confirmed federated learning as a feasible approach for data analyses that addresses the challenges of data sharing and urge for further collaborative studies including relevant prognostic factors.

## Introduction

Head and neck cancers (HNCs) include several heterogeneous types of epithelial tumors in terms of their sites of origin (i.e., tumors of the larynx, oral cavity, oropharynx, hypopharynx, major salivary glands, nasopharynx, nasal cavity, and sinuses), histological subtypes (predominantly squamous cell carcinomas, but more than 20 distinct histological subtypes may arise in this area), risk factors, incidence, and prognosis. Care for these tumors is complex, especially when diagnosis is late in the advanced stage, and often requires a multidisciplinary approach, which is best delivered in expert centers (1).

In Europe, all HNCs are rare and their 5-year survival was <60% in 2000–2007 (2). The epidemiology of HNCs differs in East Asia. Some HNCs (i.e., oral cavity and oropharynx) are less rare and carry a better prognosis than in Europe (2–5).

Survival differences among countries can be explained by different stage at diagnosis and different access to and/or quality of treatment. An important role may also be played by the different distribution and survival of the anatomical site and subsites of the cancer (2). In Europe, the adjustment for subsites narrowed the difference between countries (2). The site of origin of HNC is a major determinant of prognosis, because of both the different stage at diagnosis and the different surgical treatment options. The subsites are also a major factor (e.g., cancer of the tonsil has a better prognosis than cancers developing in other parts of the oropharynx; supraglottic cancer has a worse prognosis than glottic cancer etc.) (6).

Against this background, our objective was to describe HNCs survival in Europe and Asian countries.

We leveraged RARECAREnet Asia, a collaboration between European and selected Asian population-based cancer registries (PBCRs), namely, Taiwan, Korea, and Japan, initiated in the context of Rare Cancers Asia (https://www.rarecancerseurope.org/rare-cancers-asia), with the aim of learning from each other by considering differences in cancer epidemiology in Europe and Asia.

## Materials and methods

### Data

Our study includes first and subsequent malignant epithelial HNC diagnosed in men and women. We included the oral cavity, hypopharynx, larynx, nasal cavity, paranasal sinuses, major salivary gland, oropharynx, and nasopharynx (**Appendix Table A**).

European cases were provided by 94 PBCRs from 27 EU countries contributing to RARECAREnet (http://rarecarenet.istitutotumori.mi.it/rarecarenet/). Asian cases were provided by the national PBCRs of Taiwan and Japan. The Korean CR did not join this study. RARECAREnet PBCRs cover 46% of the European Union population (excluding Norway, Switzerland, and Iceland, which are not EU members and including UK and Ireland as they were Eu members at the time of the data colection), corresponding to approximately 208 million inhabitants; Taiwan is a national PBCR covering 23 million inhabitants, and Japanese data cover 37% of the population, corresponding to approximately 50 million inhabitants.

The case diagnosis period was 2009–2011 in Japan and Taiwan and 2000–2007 in Europe; follow-up was at 31 December 2016 and 31 December 2008 for the Asian PBCRs and RARECAREnet, respectively. We classified age into five groups: 0–44, 45–54, 55–64, 65–74, and 75+ (2). We defined subsite grouping on the basis of shared risk factors (6) and/or a similar prognosis, with few exceptions. For oropharyngeal tumors, we used tonsil-related sites (TRA) and non-tonsil-related sites (nTRA) as proxies of human papillomavirus (HPV)-positive and HPV-negative sites, respectively (7). We grouped nasopharyngeal (NPC) tumors into keratinizing and non-keratinizing cancers (8) (**Appendix Table A**).

### Data quality

We performed systematic data checks according to International Association of Cancer Registries (IACR) and International Agency for Research on Cancer (IARC) rules, together with standard and rare-cancer-specific data quality indicators: the proportion of cases known from the death certificate only (DCO), the proportion of cases diagnosed incidentally at autopsy, the proportion of microscopically verified (MV) cases, the proportion of not otherwise specified (NOS) morphology, and the proportion of NOS topography (3).

For this specific analysis, we also performed common data quality checks for survival (9, 10) on the overall database and for the HNC cases.

Furthermore, we checked the proportion of NOS topography (C06.8–C06.9, C08.8–C08.9, C10.8–C10.9, C11.8–C11.9, C31.8–C31.9, and C32.9) in the different HNC sites to assess the feasibility of performing subsite analyses. In Europe, we selected all the PBCRs in which the proportion of NOS topography cases was lower than 30%. Accordingly, for the oropharyngeal cancer analyses, we excluded the PBCRs of Austria, Bulgaria, Croatia, Finland, Iceland, Latvia, Lithuania, Poland, and Portugal; for the laryngeal cancer analyses, we excluded the PBCRs of Estonia, Lithuania, and the German PBCRs of Brandenburg, Hamburg, Mecklenburg-Vorpommern, North Rhine-Westfalia, and Saxony.

### Statistical method

We estimated relative survival (RS), which is the ratio of the observed survival of cancer patients to the expected survival in the general population for the same region (or country), age, sex, and calendar year. The RS was estimated by the Ederer II method (11) with a complete approach for European data and a cohort approach for the Asian countries. Five-year RS was estimated by site, country, and subsite or histological subtype group.

Since age, sex, subsite, and NPC histologies are prognostic factors that may have a different distribution across countries, we modeled RS with a generalized linear model, which implies a Poisson distribution of the number of observed deaths in each interval (12). The model provides estimates of relative excess risk of death (RER) for Europe vs. Taiwan and Japan, considering as covariates time since diagnosis, age, sex, and anatomical subsite for cancers of the oral cavity, hypopharynx, larynx, nasal cavity, paranasal sinus, major salivary gland, or histological subtype for the oropharynx and nasopharynx. The model included the NOS subsites. However, we performed a sensitivity analysis by excluding NOS subsites from the model.

### Federated learning approach

We leveraged the Personal Health Train (PHT) concept to address issues related to data sharing. The PHT concept enables data from multiple organizations to be analyzed without identifiable data leaving the organization.

Vantage6 (https://www.vantage6.ai) is an open-source implementation of the PHT that uses the mathematical principle of “federated learning”, applied, for this study, to horizontally partitioned data (i.e., organizations provide data from different patient cohorts, but with similar characteristics/items) (13). Federated learning is based on the mathematical principle of splitting computations into parts at stations and a central part. The stations share sub-computations with the central server only and the results returned are the same as the centralized implementation (14).

In this study, the stations were the RARECAREnet database in Milan and the Asian PBCRs in Taiwan and Japan. Before performing the analyses, we developed a code in Stata to harmonize the datasets of the PBCRs contributing to the study. The code checked the format and name of each variable, generated new variables, defined the selection criteria, and produced the stratified yearly life tables used by the Poisson model to estimate the RERs.

The federated algorithm iteratively analyzed the three separate databases and returned the same results as the centralized implementation. The mathematical decomposition of the algorithm behind the generalized linear model was demonstrated by Jones and the iteratively reweighted least square algorithm was used to find approximate maximum likelihood estimates for the parameters of the model (14, 15).

We ran the federated Poisson model, which briefly means that each station iteratively computed the mathematical parameters of the model (15). At each iteration, the aggregated statistics from the stations were combined to centrally compute an updated estimation of the RERs; when the estimation of the RERs converged, the algorithm finished (16).

## Results

The PBCRs had good quality data. The percentage of DCO cases for epithelial HNC were 1.6% in Europe, 0.4% in Taiwan, and 3.2% in Japan (**Appendix Table B**). The percentage of cases discovered at autopsy were <0.2% in each of the three datasets, and the proportion of patients lost to follow-up or censored alive within 5 years of diagnosis was 0 for Japan and Taiwan and 1.6% for Europe.

**Figure 1:**
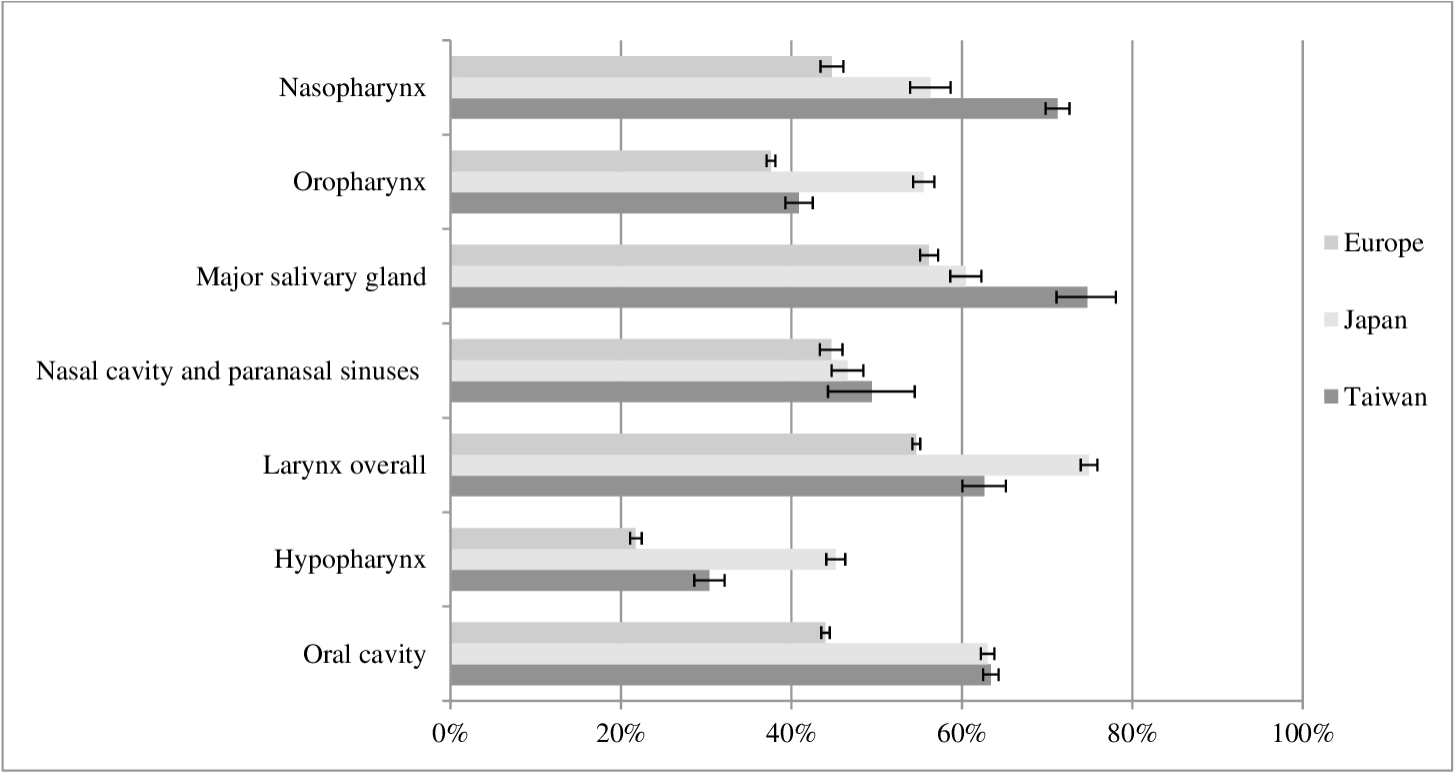
Five-year relative survival for head and neck cancers with 95% confidence intervals, by site and geographical area.

Five-year RS for HNC varied between geographical areas, with Europe having lower RS than both Japan and Taiwan for each HNC site. The largest RS difference between Europe and Taiwan was observed for NPC and between Europe and Japan for hypopharyngeal cancers. No major differences were observed for tumors of the nasal cavity and paranasal sinuses (**Figure 1**).

**Figure 2:**
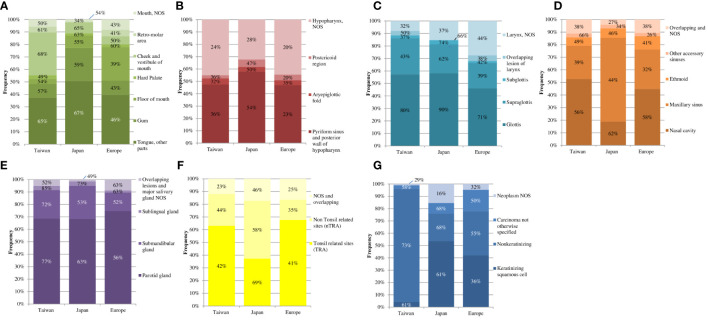
Distribution (height of the colored rectangles) together with the 5-year relative survival (reported as number% in each colored rectangle) in the different geographical areas for the subsites of the oral cavity **(A)**, hypopharynx **(B)**, larynx **(C)**, nasal cavity and paranal sinuses **(D)**, and major salivary gland **(E)** and for the histological subtype groups of the oropharynx **(F)** and nasopharynx **(G)**.

**Figure 2** shows the distribution and 5-year RS by subsites for cancers of the oral cavity, hypopharynx, larynx, nasal cavity, paranasal sinus, and major salivary gland, and by histological subtype group for the oropharynx and nasopharynx among the geographical areas.

The tongue was the most common site of oral cavity cancer in all three areas, comprising approximately 40% of patients with oral cavity cancers. However, the European RS for this subsite was 20 percentage points lower than in Taiwan and Japan. Tumors of the cheek and vestibule of the mouth were less common in Europe and Japan (<10%) and more common in Taiwan (34%). In Europe, RS was 18 and 15 percentage points lower than in Taiwan and Japan, respectively. Finally, the percentage of floor of the mouth cancers was at least three times higher (27%) in Europe than in the other two countries, also exhibiting the lowest RS.

For tumors of the hypopharynx, the distribution of each subsite was similar across the three geographical areas. However, the RS of cancers of the pyriform sinus and posterior hypopharynx in Europe was 13 percentage points lower than in Taiwan and 31 percentage points lower than in Japan.

Subsite distributions were similar among the three areas also for laryngeal cancers. About half of these patients had cancer of the glottis, with an RS ranging from 71% in Europe to 90% in Japan. Another common subsite was the supraglottis, approximately 20% in all three areas, and its RS varied from 39% in Europe to 62% in Japan.

For cancers of the nasal cavity and paranasal sinuses (i.e., maxillary, ethmoid, and other accessory sinuses), we observed no major RS differences among the three areas. However, in Japan, the maxillary sinus was the most common cancer site and also had the highest RS (44%) compared to the other areas.

Of the major salivary gland cancers, parotid gland cancers were the most common in all three geographical areas, but survival was 56% in Europe, 63% in Japan, and 77% in Taiwan.

In Europe and Taiwan, approximately 65% of oropharyngeal cancers occurred in TRA, with similar survival rates in both areas (approximately 40%). In Japan, about one-third of oropharyngeal cancers were in TRA, with an RS of 69%.

We stratified NPC by histological subtypes. In Taiwan, almost all of NPC cancers were non-keratinizing and showed the highest RS (73%). In Europe and Japan, the proportion of non-keratinizing cancers was <35%, with an RS of 55% and 68%, respectively. The proportion of keratinizing NPC was similar in Europe and Japan (approximately 50%) but RS was 61% in Japan and 36% in Europe. Taiwan had the same RS for keratinizing NPC as Japan.

There were less than 30% NOS subsites for all the sites, except for the hypopharynx. We found a more precise definition (<15% of NOS) for the oral cavity, major salivary gland, and nasal cavity. For the larynx, we observed a high percentage of NOS (27%) in Europe compared with the two Asian countries: 8% in Taiwan and 15% in Japan.

Differences between Europe and both Asian countries persisted even after adjustments for time from diagnosis, sex, age, and subsite or histological subtype for nearly all HNC sites (**Table 1**).

**Table 1:** Relative excess risk of death (RERs), with respective standard errors (SE) and *p*-values by geographical area, with Europe as reference, for each site.

Site	Geographical area	RER*	SE	*p*-value
Oral cavity	Europe	1		
	Japan	0.59	0.01	<0.0001
	Taiwan	0.66	0.01	<0.0001
Hypopharynx	Europe	1		
	Japan	0.52	0.01	<0.0001
	Taiwan	0.79	0.02	<0.0001
Larynx	Europe	1		
	Japan	0.58	0.01	<0.0001
	Taiwan	0.92	0.04	0.046
Nasal cavity and paranasal sinuses	Europe	1		
	Japan	0.85	0.03	<0.0001
	Taiwan	0.94	0.07	0.44
Major salivary gland	Europe	1		
	Japan	0.82	0.03	<0.0001
	Taiwan	0.65	0.05	<0.0001
Oropharynx	Europe	1		
	Japan	0.45	0.01	<0.0001
	Taiwan	0.93	0.02	0.0047
Nasopharynx	Europe	1		
	Japan	0.42	0.02	<0.0001
	Taiwan	0.58	0.02	<0.0001

*RERs are adjusted by time since diagnosis, age, sex, subsite (for oral cavity, hypopharynx, larynx, nasal cavity and paranasal sinuses, and major salivary gland) and histological subtype group (for oropharynx and nasopharynx).

Among all the HNC sites, we found the lowest RS differences between Europe and the Asian countries for nasal cavity cancers. After adjusting for prognostic factors, there was a 6% lower risk of death in Taiwan and a 15% lower risk of death in Japan (RER = 0.94, *p*-value = 0.44; RER = 0.85, *p*-value < 0.0001, respectively) compared to Europe (**Table 1**). The highest differences between Europe and the Asian countries remained for NPC. NPC patients diagnosed in Japan and Taiwan had 58% and 42% lower excess mortality than those diagnosed in Europe. The differences for the other sites were intermediate although all RERs were statistically significant (**Table 1**). The results did not change even when excluding NOS subsites from the models (data not shown).

## Discussion

Two major findings of our study were that survival for HNC in Europe lags behind the two East Asian countries, and that federated learning is a feasible approach for data analyses addressing the challenges of data sharing across organizations and geographies.

Significant differences in RS for nasal cavity and paranasal sinus cancers were observed between Europe and Japan only. In Japan, we confirmed the high percentage of maxillary sinus cancer previously reported in the literature. Possible explanations include (a) exposure to different varieties of wood dust in Japan compared to other countries; (b) a high prevalence of chronic sinusitis in the Japanese population, which has been associated with maxillary sinus squamous cell carcinoma; and (c) the effect of cigarette smoking, since a significant dose–response relationship has been reported between the number of cigarettes smoked per day and maxillary sinus squamous cell carcinoma among men living in Hokkaido, Japan (17–19). We thus hypothesized that the high incidence of maxillary sinus cancer in Japan had strengthened expertise in the diagnosis and treatment of these cancers and consequently of related nasal cavity and paranasal sinus malignancies.

The lower RS in Europe for salivary gland tumor subsites could be due to the different distribution of squamous cell tumors, which were more common in Europe (19%) than in Taiwan and Japan (5% and 8%, respectively) (**Appendix Table C**). Squamous cell tumors of the salivary gland are mainly skin metastases in the parotid gland, which are associated with a poor prognosis (20).

RS differences for oropharyngeal cancers remained also after the attempt to adjust for HPV-related sites. However, the low RS of TRA cancers in Europe and Taiwan and the relatively high RS of nTRA cancers in Japan suggest that the sites used as a proxy for HPV-related cancers may have been misclassified. In the period of diagnosis included in our study, the dedicated WHO morphology codes differentiating HPV-related and unrelated squamous cell carcinoma had not yet been issued, whereas the ICD-O-3.2 version (8085/3 and 8086/3) is now available. No major improvements for larynx cancer treatment have been made in recent decades. We thus hypothesized that early diagnosis could contribute to explaining the observed differences in RS. The high survival rates in Japan for hypopharyngeal cancer could also be attributed to the early diagnosis of a high proportion of hypopharyngeal tumors following incidental discovery during screening for the early diagnosis of stomach and esophageal cancers. Finally, NPC and oral cavity cancers are endemic in Taiwan and so high expertise and early diagnosis could contribute to explaining the high RS. It is worth mentioning that Taiwan has an oral cancer prevention plan that includes the promotion of regular dental examinations to ensure timely diagnosis and intervention (21). In addition, Taiwan has a comprehensive national health insurance system that provides coverage for oral health services. This allows people to undergo preventive care in a timely manner, including dental examinations, oral cancer screening, and treatment if needed. In addition, since 1999, oral cancer screening has been offered in Taiwan to all smokers and betel quid chewers over the age of 18.

The high survival in Japan has already been reported by other international studies (10). It has been hypothesized that, since the number of clinics equipped with CT and endoscopy is higher in Japan than in other countries, the medicalization of even mild symptoms may have contributed to early diagnosis and thus high survival.

The different analysis periods could also be very relevant. The European population was treated before 2007, when intensity-modulated radiotherapy (IMRT) was not yet part of the standard of care. Moreover, the use of different induction chemotherapy regimens in the two periods may be another reason for the different survival rates in the two populations (22–24).

The increase in RS in Europe from 1999–2001 to 2005–2007 for all HNCs, except for laryngeal cancer, reflects improvements in diagnosis, staging, and treatment for HNC. Nevertheless, multimodality-based management of HNC is becoming increasingly complex, especially for advanced-stage patients. In Europe, in the first decade of the 2000s, more than half of HNC patients were diagnosed at an advanced stage (regional or metastatic) in each head and neck site (1, 2). The high percentage of advanced-stage HNC at diagnosis could be a major contributing factor to the observed RS. Furthermore, in some European countries (e.g., Norway, Finland, Sweden, The Netherlands, and Italy), survival was significantly better than the European average (2). In Europe, the heterogeneity of site distribution only partially contributed to explaining differences in RS among countries (2). The possible causes of these observations are access to treatment and its quality, leading to lower survival in Europe as a whole (2, 25).

Our study has some limitations. Even if stage and treatment are important prognostic factors, this information was not available in the DB used for our study. Although Taiwan and Japan registered stage at diagnosis and treatment, we were unable to consider these covariates in the analysis because they were not routinely collected by all CRs in Europe (2). Therefore, we could only speculate on their contribution to explaining the observed survival differences. Information on patients’ race was not collected in the European data, but as the percentage of foreign-born population in the EU is only 8.5%, we do not expect this missing item to affect the results (26).

We analyzed different periods of diagnosis based on the availability of each dataset. Accordingly, we used a complete approach for European data and a cohort approach for the Asian countries to provide more comparable data. We chose the cohort approach for the Asian data to avoid including the most recently diagnosed cases that would have further increased the time gap between the estimates.

It took a long time to develop and apply the Stata code for data standardization. This suggests that innovative solutions for data standardization (e.g., OMOP CDM) should be promoted to ensure interoperability and reusability of data.

Our study also has several strengths. Our study exploited the large database of cancer cases collected by RARECAREnet—the largest cooperative study on population-based rare cancer survival in Europe—and PBCRs of Taiwan and Japan. We applied a standard case collection protocol and uniform quality control procedures to all datasets. As mortality from causes other than the relevant cancer can vary widely among geographical areas, we estimated 5-year relative survival: a standard indicator for comparing cancer survival in population-based settings. This unique collaboration also fostered the standardization of data collection across PBCRs.

We reported lower RS in Europe compared to Taiwan and Japan. These descriptive results are a starting point to stimulate more analytical studies to properly interpret RS differences in HNC across continents and to understand how to improve HNC survival in Europe. Indeed, further studies including additional information on stage, treatment, and socioeconomic status are warranted. Collaborative intercontinental studies are essential especially considering that these cancers are rare to generate hypotheses on possible different risk factors and different preventive and treatment strategies, increasing knowledge on such rare cancers.

This was the first time the VANTAGE6 platform was used to perform analyses on three different nodes involving PBCR data. The successful outcome of this analysis highlights the power of the federated learning, which, at this point, can be considered extendable to numerous nodes.

## Data availability statement

The original contributions presented in the study are included in the article/**Supplementary Material**, further inquiries can be directed to the corresponding author.

## Author contributions

AT, GGe, CJC, and TM: concept of the study; LB, AT, GGe, CJC, and TM: design of the study; LB, AT, GGe, CJC, HC, and TM: preparation of the data; LB, SB, AG, FM, and MC: performed the analysis; LB, AT, CJC, TM, AG, RM-G, MG, JR-C, LL, SCr, CR, SCa, MM, and GGa: interpretation of results; LB and AT: draft the article; all authors: critically reviewed the article for important intellectual content. The work reported in the paper has been performed by the authors, unless clearly specified in the text. All authors contributed to the article and approved the submitted version.

## Group member of RARECAREnet working group

The full list of the members of the RARECAREnet working group can be found in the Supplementary Materials.
