# Supplementary material for: Head and neck cancers survival in Europe, Taiwan, and Japan: results from RARECAREnet Asia based on a privacy-preserving federated infrastructure
Source: Front Oncol. 2023 Sep 13;13:1219111. doi: 10.3389/fonc.2023.1219111 (PMC10534949; doi:10.3389/fonc.2023.1219111)
Supplement: Supplementary file 1 [file DataSheet_1.pdf]

APPENDIX

Table of Contents:

- Page 2,3: Appendix Table A “Definition of head and neck sites and histologies included in the study, based on the topography and morphology code of the International Classification for Disease for Oncology (ICD-O), Third Revision. Definition of subsites for cancer of the oral cavity, hypopharynx, larynx, nasal cavity, salivary gland cancer, and definition of histological subtype groups for oropharyngeal and nasopharyngeal cancers.”
- Page 4: Appendix Table B “Data quality indicators for all cancers and for malignant epithelial head and neck cancer patients by geographical area.”
- Page 5: Appendix Table C ”Distribution of squamous cancers occurring in major salivary gland tumours and in subsites by geographical area.”
- Page 6: Appendix Table D “RARECAREnet WG.”

Table A: Definition of head and neck sites and histologies included in the study, based on the topography and morphology code of the International Classification for Disease for Oncology (ICD-O), Third Revision. Definition of subsites for cancer of the oral cavity, hypopharynx, larynx, nasal cavity, salivary gland cancer, and definition of histological subtype groups for oropharyngeal and nasopharyngeal cancers.

Site (ICD-O3 code)	Sub-Site/Histology group	ICD-O3 code: sub-site/histology group	ICD-O3 code: Histology
**Oral cavity (C02.0-C02.3, C02.9, C03.0-C05.0, C06.0-C06.9)**	Tongue, other parts	C02.0–C02.3, C02.9	8000, 8001, 8003-8004, 8010- 8012, 8015, 8020-8022, 8030-8034, 8050-8052, 8070-8078, 8082-8084, 8123, 8560, 8951, 8980, 8981; 8140, 8141, 8143, 8144, 8147, 8190, 8200, 8201,8211, 8230, 8231, 8260, 8261, 8263, 8290, 8310, 8320, 8323, 8401, 8430, 8440, 8450, 8480-8500, 8504, 8510, 8525, 8550, 8551, 8562, 8570, 8574, 8575, 8941, 8982
	Gum	C03.0–C03.9	
	Floor of mouth	C04.0–C04.9	
	Hard palate	C05.0	
	Cheek and vestibule of mouth	C06.0–C06.1	
	Retromolar area	C06.2	
	Mouth, NOS	C06.8–C06.9	
**Hypopharynx (C12.9, C13)**	Pyriform sinus and posterior wall of hypopharynx	C12.9, C13.2	8000, 8001, 8003-8005, 8010-8012, 8020-8022, 8030-8033, 8035, 8050-8052, 8070-8078, 8082-8084, 8120, 8123, 8560, 8951, 8980; 8140, 8141, 8143, 8144, 8147, 8190, 8200, 8201,8211, 8230, 8231, 8260, 8261, 8263, 8290, 8310, 8320, 8323, 8401, 8430, 8440, 8450, 8480-8500, 8504, 8510, 8525, 8550, 8551, 8562, 8570, 8574, 8575, 8941, 8982
	Aryepiglottic fold	C13.1	
	Postcricoid region	C13.0	
	Hypopharynx, NOS	C13.8–C13.9	
**Larynx (C32)**	Glottis	C32.0	8000, 8001, 8003-8005, 8010-8012, 8020-8022, 8030-8033, 8035, 8050-8052, 8070-8076, 8078, 8082,-8084, 8120, 8123, 8560, 8951, 8980; 8140, 8141, 8143, 8144, 8147, 8190, 8200, 8201,8211, 8230, 8231, 8260, 8261, 8263, 8290, 8310, 8320, 8323, 8401, 8430, 8440, 8450, 8480-8500, 8504, 8510, 8525, 8550, 8551, 8562, 8570, 8574, 8575, 8941, 8982
	Supraglottis	C32.1	
	Subglottis	C32.2	
	Overlapping lesion of larynx	C32.8	
	Larynx, NOS	C32.9	
**Nasal cavity and paranal sinuses (C30.0, C31)**	Nasal cavity	C30.0	8000, 8001, 8003, 8004, 8010,-8012, 8020-8023, 8030, 8031-8034, 8046, 8050-8052, 8070-8076, 8078, 8082-8084, 8120-8123, 8130, 8560, 8980, 8140, 8141, 8143, 8144, 8147, 8190, 8200, 8201,8211, 8230, 8231, 8260,
	Maxillary sinus	C31.0	

	Ethmoid	C31.1	8261, 8263, 8290, 8310, 8320, 8323, 8401, 8430, 8440, 8450, 8480-8500, 8504, 8510, 8525, 8550, 8551, 8562, 8570, 8574, 8575, 8941, 8982
	Other accessory sinuses	C31.2, C31.3	
	Overlapping and NOS	C31.8, C31.9	
**Major salivary gland (C07.9, C08)**	Parotid gland	C07.9	8000, 8001, 8003-8005, 8010-8012, 8014, 8015, 8020,-8022, 8030,8031, 8032, 8033, 8034, 8035, 8046, 8050, 8051, 8052, 8070, 8071, 8072,8073, 8074, 8075, 8076, 8078, 8082, 8083, 8084, 8120-8123, 8130, 8140, 8141, 8143, 8144, 8147, 8190, 8200, 8201, 8211, 8230, 8231, 8255, 8260, 8261, 8262, 8263, 8290, 8310, 8320, 8323, 8401, 8410, 8430, 8440, 8450, 8480-8500, 8502,8504, 8510, 8525, 8550, 8551, 8560, 8561, 8562, 8570, 8574,8575, 8940, 8941, 8951, 8980, 8981, 8982
	Submandibular gland	C08.0	
	Sublingual gland	C08.1	
	Overlapping lesions and major salivary gland NOS	C08.8,C08.9	
**Oropharynx (C01.9, C02.4, C09.0, C09.1, C09.9, C05.1, C05.2, C10.2-C10.3, C10.8-C10.9)**	TRA	C01.9,C02.4, C09.0, C09.1, C09.9	8000, 8001, 8003-8005, 8010-8012, 8020-8022, 8030-8033, 8050-8052, 8070-8076, 8078, 8082-8086, 8120, 8121, 8123, 8560, 8951, 8980, 8140, 8141, 8143, 8144, 8147, 8190, 8200, 8201,8211, 8230, 8231, 8260, 8261, 8263, 8290, 8310, 8320, 8323, 8401, 8430, 8440, 8450, 8480-8500, 8504, 8510, 8525, 8550, 8551, 8562, 8570, 8574, 8575, 8941, 8982
	nTRA	C05.1,C05.2,C10.2, C10.3	
	NOS and overlapping	C10.8, C10.9	
**Nasopharynx (C11)**	Keratinizing squamous cell	8070, 8071	8000, 8001, 8003, 8005, 8010, 8020,8021, 8082, 8070-8073, 8083*
	Nonkeratinizing	8020,8021, 8082, 8072, 8073	
	Carcinoma not otherwise specified	8010	
	Basaloid squamous cell carcinomas*	8083*	
	Neoplasm NOS	8000-8005	

* deleted from the analysis as fewer than 0.2% of all nasopharynx cases in each country

Table B: Data quality indicators for all cancers and for malignant epithelial head and neck cancer patients by geographical area

Cancers	Geographical area	Cases	Quality indicators		
			DCO % (N/D)	Autopsy % (N/D)	% Lost to follow-up* (N/D)
All malignant cases	Japan	759,269	6.4 (48,355/759,269)	0.05 (410/759,269)	0
	Taiwan	277,833	1 (2,774/277,833)	0	0
	Europe	8,332,750	3.2 (264,936/8,332,750)	0.4 (35,529/8,332,750)	1.4 (57,952/4,100,011)
Epithelial H&N malignant tumours	Japan	20,570	3.2 (654/20,570)	0.005 (1/20,570)	0
	Taiwan	27,151	0.4 (103/27,151)	0	0
	Europe	262,645	1.6 (4,339/262,645)	0.2 (440/262,645)	1.6 (2,212/137,536)

*Lost to follow-up: cases with a potential follow-up of five years that were censored earlier

Table C: Distribution of squamous cancers occurring in major salivary gland tumours and in subsites by geographical area

Site/Subsite	Geographical area	% Squamous cancers* (N/D)
Major salivary gland tumours	Europe	19 (3381/18,254)
	Taiwan	5 (13/805)
	Japan	8 (301/3,589)
Parotid gland	Europe	19 (2,558/13,591)
	Taiwan	5 (25/554)
	Japan	8 (202/2,455)
Submandibular gland	Europe	18 (516/2,797)
	Taiwan	7 (12/186)
	Japan	10 (96/955)
Sublingual gland	Europe	29 (89/311)
	Taiwan	0 (0/26)
	Japan	2 (2/125)
Major salivary gland tumours NOS	Europe	14 (218/1,555)
	Taiwan	15 (6/39)
	Japan	2 (1/54)

*defined using the malignant morphology codes: ICD-O-3: 8070-8072

Table D: RARECAREnet Working Group

Monika Hackl (Austrian National Cancer Registry); Elizabeth Van Eycken, Kris Henau (Belgian Cancer Registry); Nadya Dimitrova, Zdravka Valerianova (Bulgaria Cancer Registry); Mario Sekerija (Croatian Cancer Registry); Ladislav Dušek (Czech National Cancer Registry); Margit Mägi (Estonian Cancer Registry); Keiu Paapsi (National Institute for Health Develpoment, Estonia); Nea Malila, Maarit Leinonen (Finnish Cancer Registry); Michel Velten (Bas Rhin Cancer Registry); Anne-Valérie Guizard (Calvados, Registre Gèneral des Tumeurs); Anne-Sophie Woronoff (Doubs Cancer Registry); Michel Robaszkiewicz (Finistère Digestive Tract Registry); Isabelle Baldi (Gironde CNS Tumour Registry); Brigitte Tretarre (Hérault Cancer Registry); Marc Colonna (Isère Cancer Registry); Florence Molinié, Anne Cowppli-Bony(Loire-Atlantique/Vendée Cancer Registry); Simona Bara (Manche Cancer Registry); Bénédicte Lapôtre-Ledoux (Somme Cancer Registry); Laetitia Daubisse-Marliac (Tarn Cancer Registry); Roland Stabenow (Berlin; Brandenburg; Mecklenburg-West Pomerania; Saxony; Saxony-Anhalt; Thüringen Cancer Registry); Sabine Luttmann (Bremen Cancer Registry, Leibniz Institute for Prevention Research and Epidemiology); Alice Nennecke (Hamburg Cancer Registry); Jutta Engel, Gabriele Schubert-Fritschle (Munich Cancer Registry); Jan Heidrich (North Rhine-Westphalia Cancer Registry); Bernd Holleczek (Saarland Cancer Registry); Jón Gunnlaugur Jónasson, Helgi Birgisson (Icelandic Cancer Registry); Kerri Clough-Gorr, Harry Comber (National Cancer Registry Ireland); Guido Mazzoleni (Alto Adige Cancer Registry); Adriano Giacomin (Biella Cancer Registry); Antonella Sutera Sardo (Catanzaro Cancer Registry); Adele Caldarella, Teresa Intrieri (Tuscany Cancer Registry); Luigino Dal Maso, Martina Taborelli (Friuli Venezia Giulia Cancer Registry, Centro di Riferimento Oncologico di Aviano (CRO) di Aviano, IRCCS); Roberta De Angelis, Silvia Francisci, Corrado Di Benedetto, Sandra Mallone, Daniela Pierannunzio, Andrea Tavilla, Silvia Rossi, Mariano Santaquilani (Istituto Superiore di Sanità, Rome); Fabio Pannozzo (Latina Cancer Registry); Paolo Ricci (Mantova Cancer Registry); Antonio Giampiero Russo, Andreano Anita (Registro Tumori dell'ATS di Milano); Gianbattista Spagnoli (Modena Cancer Registry); Mario Fusco (Napoli 3 South Cancer Registry); Mario Usala (Nuoro Cancer Registry); Francesco Vitale (Palermo Cancer Registry); Maria Michiara (Parma Cancer Registry); Rosario Tumino, Giuseppe Cascone (Ragusa Cancer Registry); Lucia Mangone (Reggio Emilia Cancer Registry); Fabio Falcini (Romagna Cancer Registry, Istituto Scientifico Romagnolo per lo Studio e la Cura dei Tumori (IRST), IRCCS, Meldola, Forlì, Italy-Azienda Usl della Romagna, Forlì, Italy); Rosa Vattiato (Romagna Cancer Registry); Stefano Ferretti (Ferrara Cancer Registry); Rosa Angela Filiberti, Enza Marani (RT Liguria, Ospedale Policlinico San Martino, Genova); Arturo Iannelli, Anna Luisa Caiazzo (Salerno Cancer Registry); Rosaria Cesaraccio (Sassari Cancer Registry); Silvano Piffer, Maria Gentilini (Servizio Epidemiologia Clinica e Valutativa, Trento); Anselmo Madeddu, Antonino Ziino Colanino (Siracusa Cancer Registry); Anna Clara Fanetti (Sondrio Cancer Registry); Pina Candela (Trapani Cancer Registry); Fabrizio Stracci (Umbria Cancer Registry); Giovanna Tagliabue (Varese Province Cancer Registry, Fondazione IRCCS Istituto Nazionale dei Tumori); Massimo Rugge (Azienda Zero, SER - Registro Tumori del Veneto); Paolo Baili, Laura Botta, Riccardo Capocaccia, Roberto Foschi, Gemma Gatta,Pamela Minicozzi, Annalisa Trama, Carmen Tereanu, Milena Sant (Fondazione IRCCS, National Cancer Institute, Milan); Santa Pildava (Latvian Cancer Registry); Giedre Smailyte (Lithuanian Cancer Registry); Neville Calleja, Dominic Agius (Malta National Cancer Registry, Health Information and Research); Tom Børge Johannesen (Norwegian Cancer Registry); Jadwiga Rachtan (Cracow Cancer Registry); Paweł Macek, Stanisław Góźdź (Kielce Cancer Registry); Jerzy Błaszczyk, Kamila Kępska (Lower Silesia Cancer Registry, Wroclaw); Gonçalo Forjaz de Lacerda (Açores Cancer Registry); Maria José Bento (Northern Portugal Cancer Registry); Ana Miranda (Southern Portugal Cancer Registry); Chakameh Safaei Diba (Slovakian National Cancer Registry); Vesna Zadnik, Tina Žagar (Slovenian Cancer Registry); Enrique Almar (Albacete Castilla-La Mancha Cancer Registry); Nerea Larrañaga, Arantza Lopez de Munain (Basque Country Cancer Registry, CIBERESP); José María Díaz García (Cuenca Cancer Registry); Rafael Marcos-Gragera (Girona Cancer Registry); Maria Josè Sanchez (Granada Cancer Registry , CIBERESP, ibs.Granada); Mª Dolores Chirlaque, Diego Salmerón (Murcia Cancer Registry, CIBERESP, IMIB-Arrixaca, Murcia University); Eva Ardanaz, Marcela Guevara (Navarra Cancer Registry, CIBERESP); Jaume Galceran, Marià Carulla (Tarragona Cancer Registry); Mohsen Mousavi (Basel Cancer Registry); Christine Bouchardy (Geneva Cancer Registry); Silvia M. Ess (Grisons-Glarus, St. Gallen Cancer Registry); Andrea Bordoni (Ticino Cancer Registry); Isabelle Konzelmann (Valais Cancer Registry); Jem Rashbass (Public Health England);

Damien Bennet, Sinead Hawkins (Northern Ireland Cancer Registry); David H Brewster (Scotland Cancer Registry); Dyfed Wyn Huws (Welsh Cancer Intelligence and Surveillance Unit); Otto Visser, Jan Maarten van der Zwan, Sabine Siesling (The Netherlands Cancer Registry); Ellen Benhamou (Institut de Cancérologie Gustave Roussy, Villejuif, France); Renèe Otter (Scientific Institute of Public Health, Brussels).
